# GENDER DIFFERENCES IN DEPRESSIVE SYMPTOMS OF PATIENTS WITH END-STAGE LIVER DISEASE

**DOI:** 10.1093/geroni/igz038.2119

**Published:** 2019-11-08

**Authors:** Shirin O Hiatt, John Lee, Lissi Hansen

**Affiliations:** 1 Oregon Health & Science University School of Nursing, Portland, Oregon, United States; 2 Oregon Health & Science University, Portland, Oregon, United States

## Abstract

Background/aims: Similar to many chronic diseases, depression is common in patients with end-stage liver disease (ESLD), although gender differences are less known. Understanding the burden of depression in this population may help identify at-risk patients who would benefit from early intervention. The purpose of this work, therefore, is to describe gender differences in depressive symptoms in patients with ESLD. Methods: Patient data were collected as part of a larger study (NINR: 1R01NR016017-01). Patients (≥ 21 years) diagnosed with ESLD from the outpatient hepatology clinics of two healthcare systems in Pacific Northwest completed Patient Health Questionnaire (PHQ-9). Survey data were analyzed using descriptive statistics. Results: Sample included 154 participants, 101 males (65.6%), average age 57 years (SD=10.92), and 53 females (34.4%), average age 55 years (SD=11.28). More than 75% of the sample (78% females and 77% males) reported at least mild depression (PHQ score ≥ 5); mean PHQ-9 scores were higher for males (M=9.26±5.86) than females (M=9.10±5.07), but were not statistically different (U=2396, p=0.99). There was no significant relationship between depression severity and gender [X2 (4, N=147)=1.90, p=0.594]. Conclusion: Our study showed a high prevalence of depression in patients. A higher percentage of females reported mild to moderate depression and had higher clinically significant levels of depression (PHQ-9 score ≥ 10) than males, indicating females may be at a greater risk for depression. Females may, therefore, gain greater benefit from interventions to improve depressive symptoms. Future studies should examine the benefit of interventions on depression severity in this patient population.

